# Highly pathogenic porcine reproductive and respiratory syndrome virus nonstructural protein 1 interacts with TRAF6 to activate the TAK1/p38/JNK/AP-1 signaling and induce IL-1β

**DOI:** 10.1128/jvi.00428-26

**Published:** 2026-06-12

**Authors:** Jiaying Zhu, Shuyuan Guo, Ailing Liang, Chang Liu, Hao Zhou, Zhongzhou Chen, Wen-hai Feng

**Affiliations:** 1 Frontiers Science Center for Molecular Design Breeding, Beijing, China; 2 State Key Laboratory of Animal Biotech Breeding, Beijing, China; 3 Ministry of Agriculture Key Laboratory of Soil Microbiology, Beijing, China; 4 Department of Microbiology and Immunology, College of Biological Sciences, China Agricultural University 34752, Beijing, China; Loyola University Chicago - Health Sciences Campus, Maywood, Illinois, USA

**Keywords:** PRRSV, nsp1, IL-1β, inflammation, MAPK pathways

## Abstract

Porcine reproductive and respiratory syndrome (PRRS) caused by PRRS virus (PRRSV) is a highly contagious disease that severely threatens the global pig industry. A significant symptom of PRRS is severe pneumonia. At present, studies have shown that IL-1β is upregulated by PRRSV infection *in vivo* and *in vitro*, while the underlying mechanism remains unknown. Here, we explored how PRRSV regulated IL-1β production. We found that HP-PRRSV nsp1 upregulated the expression of IL-1β. Overexpression of nsp1 induced IL-1β production in a dose-dependent manner. Subsequently, we demonstrated that nsp1 enhanced IL-1β expression by activating JNK/p38 signaling pathways, as the addition of p38 and JNK inhibitors reduced IL-1β production induced by nsp1. Moreover, deleting the AP-1 binding motif in porcine IL-1β promoter impaired IL-1β expression induced by nsp1, suggesting that AP-1 is essential for IL-1β production. In addition, we showed that TRAF6 was required for nsp1 to activate the TAK1/JNK/p38/AP-1 signaling pathway by interacting with each other. Truncation and deletion mutation analysis indicated that R308 and R375 amino acids were important for nsp1 to interact with TRAF6 and induce IL-1β production. Finally, we constructed the recombinant viruses mutated at R308 and R375 and found that the mutated virus PRRSV-R308/375A exhibited a significantly reduced replication rate and impaired the ability to induce IL-1β compared to the wild-type strain. These findings provide new insights into the molecular mechanisms of IL-1β production induced by PRRSV and help us further understand the pathogenesis of PRRSV infection.

**IMPORTANCE:**

Highly pathogenic porcine reproductive and respiratory syndrome virus (HP-PRRSV) has been prevalent in China since 2006, causing severe pneumonia in pigs. IL-1β is a proinflammatory cytokine secreted by various cells. In this study, we demonstrated that PRRSV protein nsp1 interacted with TRAF6 to activate the TAK1/JNK/p38/AP-1 signaling pathway, consequently inducing the expression of IL-1β. Additionally, our findings revealed that R308 and R375 in nsp1 were critical amino acid residues for nsp1 to interact with TRAF6 and induce IL-1β production. Notably, the recombinant virus PRRSV-R308/375A exhibited a significantly reduced replication rate and impaired ability to induce proinflammatory cytokines *in vitro* compared to the wild-type strain HP-PRRSV. This study helps us further understand HP-PRRSV pathogenesis and provides a new idea for the development of PRRSV vaccines.

## INTRODUCTION

Porcine reproductive and respiratory syndrome (PRRS) is a severe infectious disease that threatens the pig industry worldwide. The pathogen causing PRRS is porcine reproductive and respiratory syndrome virus (PRRSV) (1, 2). PRRSV is an enveloped, single-stranded, positive-sense RNA virus, which belongs to the *Arterivirus* genus, *Arteriviridae* family, and *Nidovirales* order (3). The PRRSV genome is approximately 15.4 kb in length and contains at least 11 open-reading frames, encoding 8 structural proteins and at least 16 non-structural proteins: nsp1α, nsp1β, nsp2TF, nsp2N, nsp2-6, nsp7α, nsp7β, and nsp8-12 (4–6). In 2006, highly pathogenic PRRS (HP-PRRS) characterized by high fever, high morbidity, and high mortality emerged in southwestern China. This outbreak has rapidly spread across Asian countries, causing significant damage to swine industries (7). Up to now, it remains one of the prevalent strains in China.

HP-PRRSV infection is associated with severe lung inflammation and injury. Lung edema, hemorrhage, and peribronchiolitis can be observed quickly in the infected pigs, indicating that inflammation plays an important role in the pathogenesis of HP-PRRSV (8). In addition, proinflammatory cytokines are highly expressed, especially IL-1β, IL-6, and TNFα, and a large number of inflammatory cells, including mononuclear macrophages, neutrophils, and mast cells, infiltrate the alveolar cavity (9).

IL-1β, a member of the IL-1 family, is an important proinflammatory cytokine. A broad range of cells, including monocytes, macrophages, and neutrophils, could produce IL-1β (10). IL-1β has strong proinflammatory activity and can induce various proinflammatory mediators, such as cytokines and chemokines, ultimately leading to widespread inflammatory events (11). Previous studies have shown that IL-1β is upregulated after PRRSV infection *in vitro* and *in vivo* (12). Serum IL-1β levels are significantly associated with persistent PRRSV infection (13). Compared with the classical PRRSV strain BJ-4, IL-1β is significantly upregulated in PAMs infected by the HP-PRRSV strain HN07-1 (14). These reports suggest that PRRSV infection significantly induces IL-1β expression, and IL-1β is closely related to the pathogenesis of PRRSV. Thus, investigating the molecular mechanism of IL-1β expression induced by HP-PRRSV will help us understand PRRSV pathogenesis.

In this study, we first confirmed that HP-PRRSV significantly activated IL-1β production. Then, we found that PRRSV nonstructural protein nsp1 was the PRRSV protein that efficiently induced IL-1β expression. Subsequently, we demonstrated that nsp1 activated the TAK1/JNK/p38/AP-1 signaling pathway through interacting with TRAF6, thereby inducing the expression of IL-1β. Moreover, Lys308 and Lys375 were shown to be the key amino acid sites in nsp1 that affect the interaction between nsp1 and TRAF6 and IL-1β production. Most importantly, the recombinant virus PRRSV-R308/375A exhibited a significantly reduced replication rate and impaired ability to induce inflammation in PAMs compared to its parent wild-type HP-PRRSV strain. Our study reveals the mechanism for the induction of IL-1β by HP-PRRSV and provides further information for better understanding of PRRSV pathogenesis.

## MATERIALS AND METHODS

### Cells and virus

Porcine alveolar macrophages (PAMs) were obtained from postmortem lung lavage of 8-week-old specific, pathogen-free (SPF) pigs and maintained in RPMI 1640 medium (Macgene, China) supplemented with 10% heat-inactivated fetal bovine serum (FBS; Gibco, USA) and 1% penicillin and streptomycin (Macgene, China). CRL-2843 cells, a porcine alveolar macrophage cell line, were maintained as PAMs. HEK-293T cells (ATCC CRL-3216) were cultured in Dulbecco modified Eagle medium (DMEM; Macgene, China) with 10% heat-inactivated FBS and 1% penicillin and streptomycin. All cells were cultured at 37°C in an incubator with 5% CO_2_.

The HP-PRRSV (HV isolate; PRRSV-2) was propagated and titrated on PAMs. Briefly, the viral supernatants from cell cultures were collected at different time points after HV inoculation. The viral supernatants were diluted 10-fold in complete RPMI 1640 to infect PAMs in 96-well plates. PRRSV infection was determined at 72 h postinfection using immunofluorescent staining for PRRSV N protein. Virus titer was then determined using the Reed-Muench method and expressed as the TCID50. The virus was stored at −80°C until use.

### RNA extraction and qPCR

Total RNAs were extracted from treated cells with TRIzol reagent (Mei5bio, China) according to the manufacturer’s instructions. cDNA was prepared from 100 ng of RNA using M5 First-Strand cDNA Synthesis Kit (Mei5bio, China). QPCR analysis was performed by using a ViiA 7 real-time PCR system (Applied Biosystems, USA) and SYBR green real-time PCR Master Mix (Mei5bio, China). The relative expression level was analyzed using the 2^-ΔΔCT^ method. Gene qPCR primers are listed in Table 1. All qPCR experiments were completed in triplicate.

**TABLE 1:** Quantitative RT-PCR primer sequences used in this study

Primer	Sequence (5′−3′)
GAPDH forward primer	CCTTCCGTGTCCCTACTGCCAAC
GAPDH reverse primer	GACGCCTGCTTCACCACCTTCT
PAM IL-1β forward primer	TCCAGCCAGTCTTCATTGTT
PAM IL-1β reverse primer	GATGACAGACACCATCTGCCT
CRL IL-1β forward primer	CCCAGGAAGACGGGCTT
CRL IL-1β reverse primer	TCTGCCCTGTACCCCAAC
JNK forward primer	CAGCCCTCTCCTTTAGCACA
JNK reverse primer	TGTATCCGAGGCCAAAGTCG
P38 forward primer	GATGCCAAGCCATGAGGCAAG
P38 reverse primer	AGCATCTTCTCCAGCAAGTCAA
c-Jun forward primer	GCGCCTGATAATCCAGTCCA
c-Jun reverse primer	TGGGGCATAGGAACTGGGTA
TAK1 forward primer	ATTCCAAGCCTAAACGGGGC
TAK1 reverse primer	ACTGCCCGTTGCCTGATATG
TRAF6 forward primer	CTCATCAGAGAACAGATGCCCA
TRAF6 reverse primer	GGTACAGGAGCTACTGCGAG
IRAK1 forward primer	GCAGTTGTCACGGTTTCGTC
IARK1 reverse primer	CACAAGGATGTCCAGTCGCT

### Plasmid construction and transfection

Genomic DNA was extracted from PAMs using the DNA extraction kit (TaKaRa). A 2,112-bp-length *Sus scrofa IL-1β* gene promoter was cloned and inserted into the luciferase reporter vector pGL3-Basic at the MluI and SmaI sites. The truncated mutants of the IL-1β promoter were constructed using the primers listed in Table 2. AP-1 deletion mutants were generated by the Q5 Site-Directed Mutagenesis Kit according to the manufacturer’s instructions (NEB, USA).

**TABLE 2:** Sequences of the primers used for the truncated IL-1β promoter and mutated viral proteins

Primer	Sequence (5′−3′)
P1-F	CGACGCGTCGTTTATTGAGATTCGCTTTGTGTCC
P1-R	TCCCCCGGGGGATAACACTGCGCTCTTGAG
P2-F	CGACGCGTCGGCCTCGTTTGGTGG
P3-F	CGACGCGTCGAACTAGGGTACCAGCACC
P4-F	CGACGCGTCGGTTTCCTAAACTTAAGTCCAAGG
P5-F	CGACGCGTCGTTCAGGTTACGCGCTC
P6-F	CGACGCGTCGCAAAGGCCATTCAGGGAC
P7-F	CGACGCGTCGCCTCCATATGCTGCAGG
Δ1-F	GGAGCACAAAGTTGTCAG
Δ1-R	AACAGAAATACTGGGGTTTC
Δ2-F	TTTGCAAAAAAAAAATAAAAAGTG
Δ2-R	ATATGCAAATATATGTTTCTTTCTTC
AP-1 deletion 1-F	GGAGCACAAAGTTGTCAG
AP-1 deletion 1-R	AACAGAAATACTGGGGTTTC
AP-1 deletion 2-F	TTTGCAAAAAAAAAATAAAAAGTG
AP-1 deletion 2-R	ATATGCAAATATATGTTTCTTTCTTC
K304A-F	TGTCCCTGGCGCGTACCTACAGC
K304A-R	CCATGCTTGGTTTGATAG
R308A-F	ATACCTACAGGCGAGGCTGCAAGTTAATG
R308A-R	TTGCCAGGGACACCA
P365A-F	CAATACGTCAGCGCTGGCTGGAA
P365A-R	GGCTCAACCCTGATTCTG
R375A-F	GAAGATTTTCGCGTTTGGCAGTCATAAG
R375A-R	TCATCCTTTCCAGCC
P134A-F	CATTGTCGGGGCGGTCCCTGGGG
P134A-R	GGGTACCAACGACAACCCC

Genes encoding viral proteins were amplified by PCR and then cloned into the pRK5-Flag or pRK5-HA vector. All construction vectors were confirmed by DNA sequencing. The mutants of nsp1 were constructed using the Q5 site-directed mutagenesis kit (NEB, USA) according to the manufacturer’s instructions. The primers are listed in Table 2.

All plasmids were transfected into CRL-2843 cells, PAMs, or HEK-293T cells using jetPRIME transfection reagent (Polyplus, France) according to the manufacturer’s instructions.

### siRNA knockdown

siRNAs targeting c-Jun, p38, JNK, TAK1, and TRAF6 genes (GenePharma, China) or nonspecific controls (NC) were transfected using jetPRIME transfection reagent (Polyplus, France). The efficiency of the knockdown of protein expression was assessed by qPCR.

### Luciferase reporter assays

HEK-293T cells seeded in 24-well plates were transfected with the constructed plasmids (pRL-TK, pGL3-Basic, and IL-1β promoters) using jetPRIME transfection reagent (Polyplus, France) according to the manufacturer’s protocol. Cell extracts were prepared and analyzed for firefly and *Renilla* luciferase activities using a dual-luciferase reporter assay kit (Mei5bio, China) according to the manufacturer’s instructions.

### Western blot analysis

Cells were lysed in radioimmunoprecipitation assay (RIPA) lysis buffer (CWBIO, China) with 100 U of proteinase inhibitors (CWBIO, China) and 20 μM NaF (Sigma, USA) on ice. Proteins were separated by SDS-PAGE and transferred to polyvinylidene difluoride (PVDF) membranes (Millipore, USA). Membranes were blocked with 5% skim milk in PBS for 1 h at room temperature, followed by incubation overnight at 4°C with antibodies against p-c-Jun, c-Jun, p-p38, p38, p-JNK, JNK, p-TAK1, TAK1, and TRAF6 (MCE, USA). The membranes were washed three times with PBST and then incubated with HRP-conjugated anti-rabbit/mouse secondary Ab (1:5,000; EASYBIO, China) for 2 h at room temperature. β-actin (1:1,000) was used as a loading control. Proteins were visualized using ECL detection reagents (Mei5bio, China).

### Co-immunoprecipitation

Cells were lysed by lysis buffer (50 mM Tris-HCl, pH 7.5, 150 mM NaCl, 5 mM EDTA, 1% NP-40, and 10% glycerol; Eccasen, China) containing 100 U of proteinase inhibitors and 20 mM NaF for 20 min on ice. The cell lysate was clarified by centrifugation at 12,000 × *g* for 10 min and then incubated with 20 μL of anti-FLAG M2 Affinity beads (Sigma, USA) for 4–6 h at 4°C. The beads were washed three times with lysis buffer. The precipitates were analyzed by western blotting.

### ELISA

The IL-1β protein levels in cell culture supernatants were measured using porcine IL-1β ELISA kits (JONLNBIO, China) in accordance with the manufacturer’s instructions.

### Construction strategy for infectious cDNA clones

According to the strategies as previously described (15), a full-length cDNA clone (pcDNA3.1-HV) was obtained. The full genome of HP-PRRSV isolate HV was divided into four overlapping fragments, and the first fragment that contains R308 and R375 mutation sites was cloned by Q5 Site-Directed Mutagenesis Kit (NEB, USA). These mutant fragments were then inserted into the pcDNA3.1-HV fragment by DNA Assembly Mix Plus (LABLEAD, China) to generate the full-length infectious cDNA clones with the respective mutated site.

These plasmids (pcDNA3.1-HV, pcDNA3.1-HV-R308/375A) were transfected into HEK-293T cells. At 72 h post-transfection, cell culture supernatants were inoculated on PAMs, and an immunofluorescence assay was performed for virus detection.

### Statistical analysis

Statistical analysis was performed using GraphPad Prism software, and differences were analyzed using a Student’s *t*-test. Significance is denoted in the figures as follows: *, *P <* 0.05; **, *P* < 0.01; ***, *P* < 0.001; ns, not significant.

## RESULTS

### PRRSV nsp1 induces IL-1β expression

**Fig 1:**
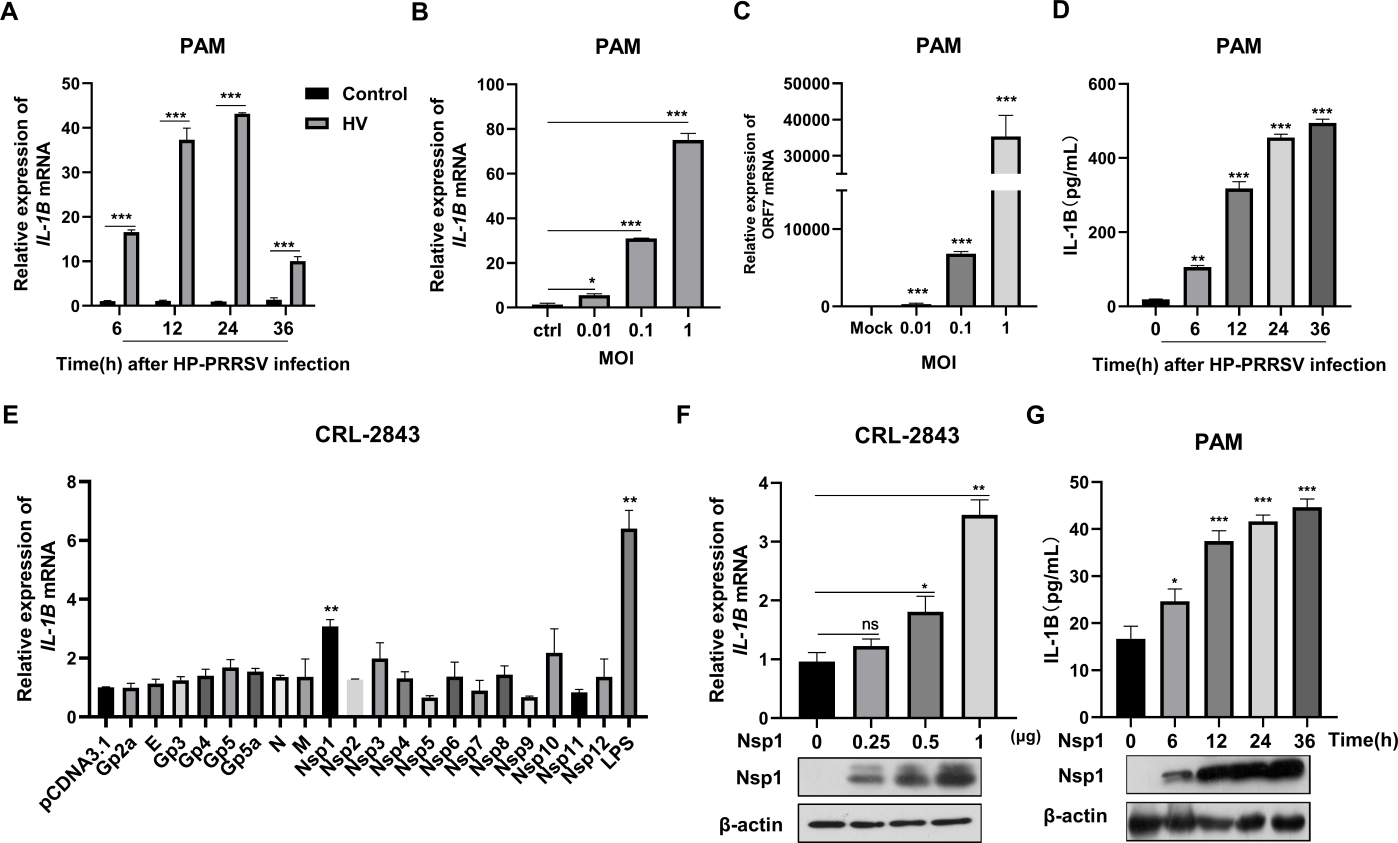
PRRSV nsp1 upregulates IL-1β production. (**A**) PAMs were inoculated with HP-PRRSV at an MOI of 0.1. Total RNAs were extracted from cell lysates at 6, 12, 24, and 36 h post-inoculation, and qPCR was used to analyze IL-1β mRNA levels. The results were normalized to GAPDH. (**B and C**) PAMs were either mock-infected or infected with HP-PRRSV at MOIs of 0.01, 0.1, and 1 for 24 h, and total RNAs were extracted for qPCR analysis to analyze the mRNA levels of IL-1β and ORF7. (**D**) Supernatants were harvested at 6, 12, 24, and 36 h after HP-PRRSV infection (MOI = 0.1) to measure IL-1β production by ELISA. (**E**) PRRSV structural and nonstructural protein expression vectors (1 μg) were transfected into CRL-2843 cells. Total RNAs were extracted at 24 h, and qPCR was used to analyze IL-1β expression; the results were expressed as the fold induction over the vector control. (**F**) CRL-2843 cells were transfected with different doses of nsp1 expression vectors, and IL-1β mRNA was analyzed at 24 h by qPCR. (**G**) Supernatants were harvested at 6, 12, 24, and 36 h after nsp1 transfection (0.5 μg) to measure IL-1β production by ELISA. The data are representative of three independent experiments (means ± SEM). Differences were evaluated by Student’s *t-*test. *, *P <* 0.05; **, *P <* 0.01; ***, *P <* 0.001; ns, not significant.

Previous studies have demonstrated that HP-PRRSV significantly upregulated the expression of IL-1β *in vitro* and *in vivo* (12). To confirm this, we infected porcine alveolar macrophages (PAMs) with HP-PRRSV strain HV and analyzed IL-1β mRNA levels using quantitative real-time PCR (qPCR) at 6 h, 12 h, 24 h, and 36 h post-infection. As shown in Fig. 1A, qPCR analysis showed that the mRNA level of IL-1β was significantly upregulated at all time points post-infection, with the highest upregulation ~40-fold at 24 h post-infection. Then, we infected PAMs with HP-PRRSV strain HV at a multiplicity of infection (MOI) of 0.01, 0.1, and 1, respectively. Our data showed that HP-PRRSV infection upregulated IL-1β in a dose-dependent manner at 24 h post-infection (Fig. 1B). Meanwhile, we detected the mRNA level of ORF7, indicating the high infection efficiency of HP-PRRSV (Fig. 1C). In addition, IL-1β in the supernatants of PAMs was measured by ELISA at 6, 12, 24, and 36 h after HP-PRRSV infection. As shown in Fig. 1D, the protein level of IL-1β was also significantly induced by HP-PRRSV. These results all verify that HP-PRRSV induces IL-1β expression.

To examine which HP-PRRSV protein(s) can induce IL-1β production, we constructed plasmids containing each of the HP-PRRSV structural and non-structural protein genes. Then, we transfected each of these plasmids into CRL-2843 cells (a cell line of porcine macrophages, the natural PRRSV targeting cell) for 24 h and examined IL-1β mRNA levels using qPCR. Our results showed that HP-PRRSV non-structural protein 1 (nsp1) significantly upregulated the mRNA level of IL-1β (Fig. 1E). Next, we transfected CRL-2843 cells with different doses (0.25, 0.5, and 1 μg) of nsp1, and the mRNA level of IL-1β was analyzed by qPCR at 24 h post-transfection. The results showed that the upregulation of IL-1β induced by nsp1 was in a dose-dependent manner (Fig. 1F). Correspondingly, the protein level of IL-1β in the supernatants was significantly elevated by nsp1 (Fig. 1G). These results indicate that HP-PRRSV nsp1 plays a crucial role in the upregulation of IL-1β.

### Nsp1 upregulates IL-1β expression through the JNK and P38 signaling pathways

**Fig 2:**
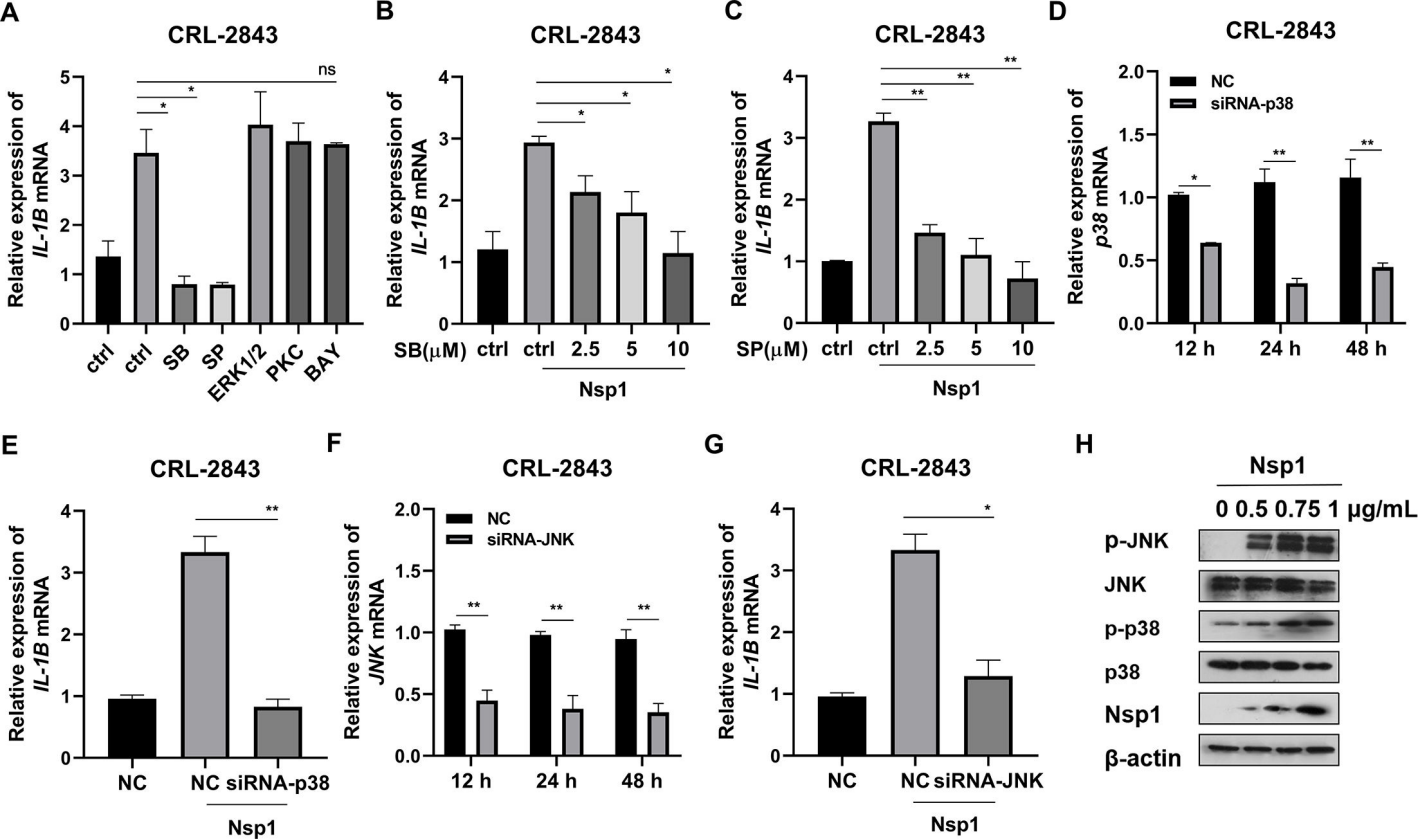
Nsp1 upregulates IL-1β expression through the JNK and p38 signaling pathways. (**A**) CRL-2843 cells were pretreated with inhibitors of p38 MAPK (SB203580, SB), JNK (SP600125, SP), ERK1/2 (HY12028, ERK1/2), PKC (PKC-IN-1, PKC), NF-κB (BAY11-7082, BAY), or DMSO (10 μM), and 1 h later, the cells were transfected with nsp1 (1 μg). After 24 h, IL-1β mRNA was analyzed by qPCR. (**B and C**) CRL-2843 cells were pretreated with p38 inhibitor (SB203580) and JNK inhibitor (SP600125) at different doses (2.5, 5, and 10 μM), and 1 h later, the cells were transfected with nsp1 (1 μg). After 24 h, the total RNAs were extracted for IL-1β mRNA analysis by qPCR. (**D and F**) CRL-2843 cells were transfected with *p38* or *JNK* siRNA (60 nM), and NC was as the control. Cells were collected at 12, 24, and 48 h after transfection, and the mRNA level of *p38* or *JNK* was detected by qPCR. (**E and G**) CRL-2843 cells were transfected with *p38* or *JNK* siRNA (60 nM), and NC was as the control. At 12 h later, cells were transfected with nsp1 (1 μg). After 24 h, cells were collected, and the mRNA level of IL-1β was analysed by qPCR. (**H**) PAMs were transfected with nsp1 at different doses (0.5, 0.75, and 1 μg/mL), and the cells were harvested at 24 h post-transfection. Western blotting was used to examine the levels of p-JNK, total-JNK, p-p38, total-p38, Nsp1 and β-actin. Differences were evaluated by Student’s *t-*test. *, *P <* 0.05; **, *P <* 0.01; ns, not significant.

To explore the mechanism underlying the enhanced production of IL-1β by nsp1, CRL-2843 cells were pretreated with selected signal transduction inhibitors for 1 h, including inhibitors for p38, JNK, PKC, ERK1/2, and NF-κB, before the transfection of the nsp1 expression plasmid. At 24 h post-transfection, cells were collected for IL-1β mRNA analysis by qPCR. The results showed that SB203580 (P38 inhibitor) and SP600125 (JNK inhibitor) significantly inhibited IL-1β expression induced by nsp1 (Fig. 2A). To further confirm the effects of p38 and JNK inhibitors, we treated CRL-2843 with p38 or JNK inhibitor at different concentrations (2.5, 5, and 10 μM) for 1 h, followed by transfection with nsp1 for 24 h. Our data showed that the inhibitory effects of both inhibitors were in a dose-dependent manner (Fig. 2B and C). To further determine the role of p38 and JNK in the induction of IL-1β by nsp1, we designed and synthesized the siRNAs for *p38* and *JNK*. We first detected the knockdown efficiency of siRNAs, and qPCR analysis indicated that p38 and JNK were specifically knocked down by siRNAs for *p38* (Fig. 2D) and *JNK* (Fig. 2F), respectively. Next, we transfected CRL-2843 cells with the siRNA for *p38* or *JNK* for 12 h, followed by transfection with nsp1 for 24 h. As shown in Fig. 2E and G, *p*38 or *JNK* silencing by siRNA led to a significant reduction in IL-1β induced by nsp1 (~75% and 61% decreases, respectively). These results suggest that nsp1-induced IL-1β expression is dependent on p38 and JNK pathways.

To examine whether nsp1 induces p38 and JNK activation, we transfected CRL-2843 cells with different amounts (0.5, 0.75, or 1 μg) of nsp1. At 24 h post-transfection, the phosphorylation levels of p38 and JNK were analyzed. Western blot analysis indicated that the phosphorylation levels of p38 and JNK were significantly increased in a dose-dependent manner (Fig. 2H), indicating that p38 and JNK are activated by nsp1.

### PRRSV nsp1-induced IL-1β expression is dependent on AP-1

**Fig 3:**
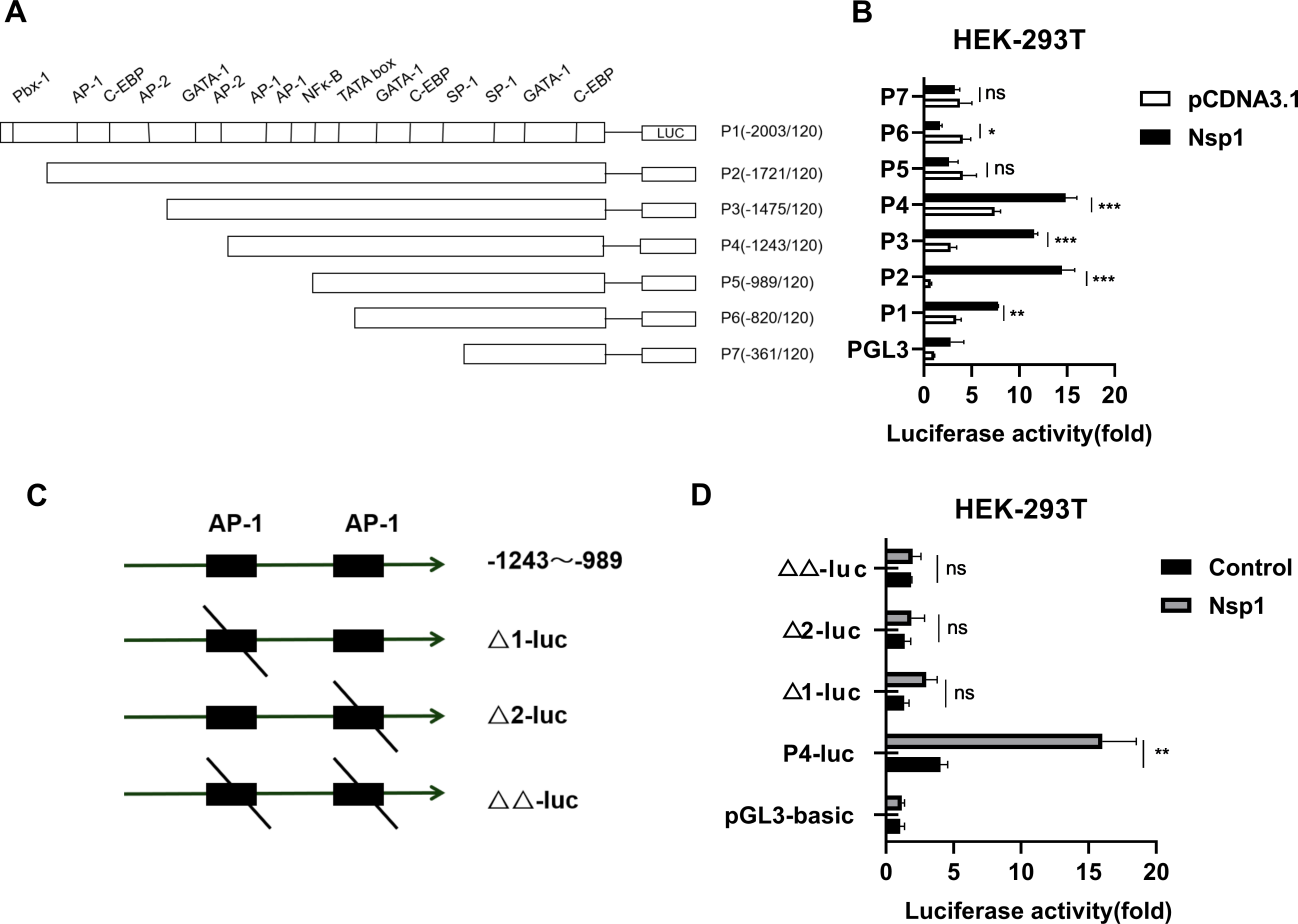
AP-1 is critical for PRRSV nsp1 to activate the IL-1β promoter. (**A**) Cloning and sequence analysis of the 2,112-bp porcine IL-1β gene 5′-flanking region. A schematic representation of the IL-1β promoter and the truncated promoter mutants. (**B**) The IL-1β promoter vector or pGL3 empty vector (0.5 μg) and nsp1 plasmid (0.5 μg) were transfected into HEK-293T cells, and the cells were then harvested to determine the luciferase activity at 24 h post-transfection. (**C**) Two AP-1 transcription factor-binding regions on the P4 promoter were deleted alternatively, and the AP-1-deleted luciferase reporter vectors were constructed: Δ1-luc, Δ2-luc, and ΔΔ-Luc. (**D**) Δ1-luc, Δ2-luc, or ΔΔ-Luc (0.5 μg) was co-transfected with nsp1 plasmid (0.5 μg) into HEK-293T cells. At 24 h post-transfection, cells were harvested to analyze the luciferase activity of the promoter. Differences were evaluated by Student's *t-*test. *, *P <* 0.05; **, *P <* 0.01; ***, *P <* 0.001; ns, not significant.

To further investigate the regulation mechanism of IL-1β production induced by nsp1, we cloned a 2,112-bp fragment of the 5′-flanking region of the porcine *IL-1β* gene. To determine the region of the porcine IL-1β promoter that responds to nsp1, we constructed a series of truncated IL-1β promoters using luciferase reporter plasmid pGL3 (Fig. 3A). Next, we transfected each of the truncated reporter plasmids with nsp1 expression plasmid into HEK-293T cells. At 24 h post-transfection, the cells were harvested for luciferase assay. Luciferase reporting analysis showed that the constructs P1–P4 exhibited higher luciferase activities. However, the constructs P5–P7 showed no significant difference when compared to the control, indicating that the key regulatory elements for nsp1 to induce IL-1β production might exist in the region between P4 and P5 (Fig. 3B). Using bioinformatics analysis (https://services.healthtech.dtu.dk/services/Promoter-2.0/) to analyze the transcription factor-binding sites, we found that there were two AP-1 sites in this region. To determine the role of the AP-1 sites in the activation of the IL-1β promoter by nsp1, we constructed single-deleted and double-deleted mutant plasmids of AP-1-binding sites, respectively (Fig. 3C). Then, we transfected each of the mutants with nsp1 expression plasmid into HEK-293T cells and examined the activation of the mutated IL-1β promoters using luciferase reporting assay. The results showed that the absence of any of the AP-1-binding sites significantly impaired the activation of IL-1β promoters by nsp1 (Fig. 3D). These findings imply that both AP-1 sites are critical for nsp1 to activate IL-1β promoters.

**Fig 4:**
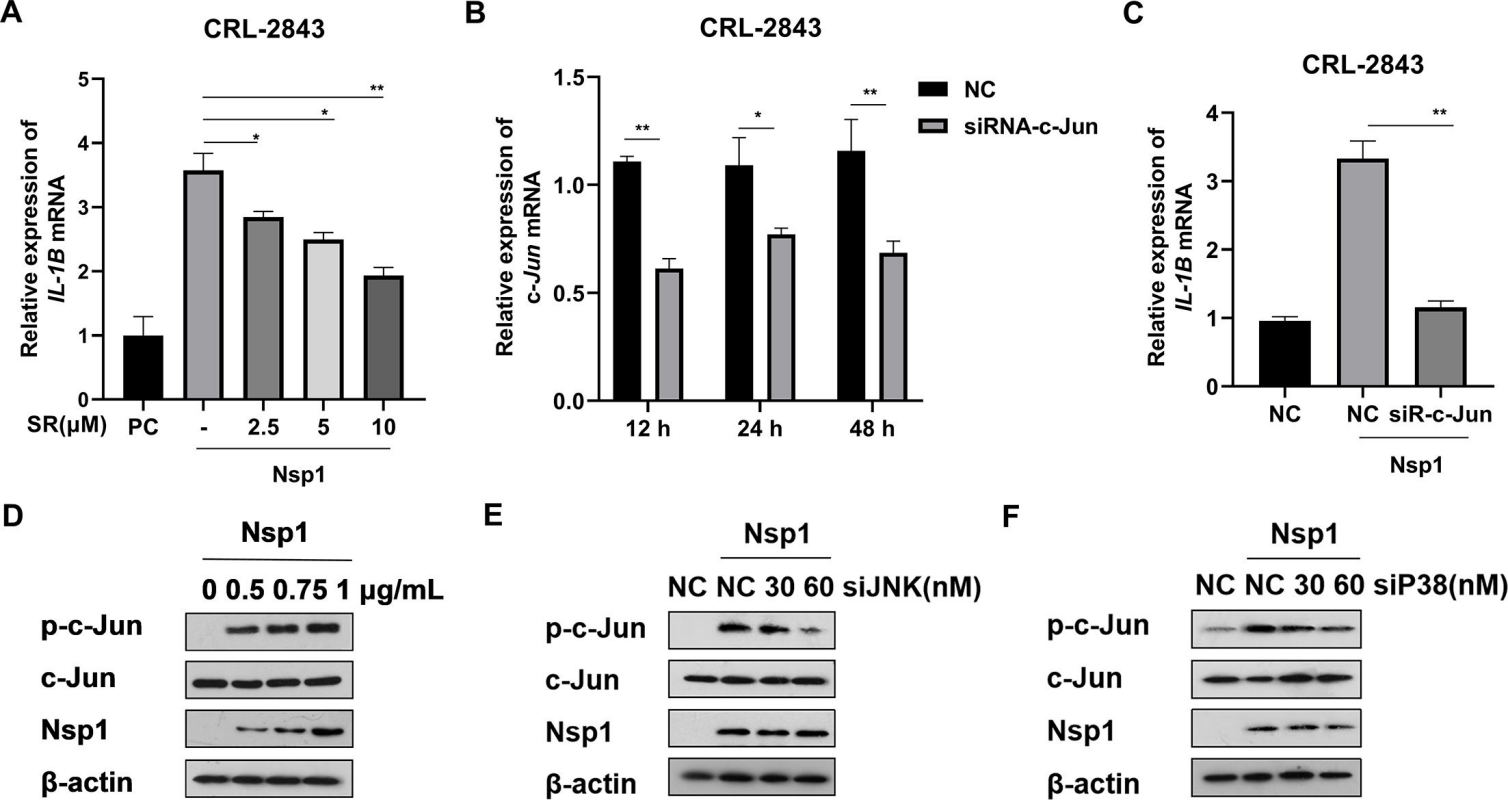
PRRSV nsp1-induced IL-1β expression is dependent on AP-1. (**A**) CRL-2843 cells were treated with AP-1 inhibitor SR-11302 at different doses (2.5, 5, and 10 μM) for 1 h, followed by transfection with the nsp1 plasmid (1 μg). Cells were collected at 24 h, and the mRNA level of IL-1β was analyzed by qPCR. (**B**) CRL-2843 cells were transfected with *c-Jun* siRNA (60 nM), with NC as the control. Cells were collected 12, 24, and 48 h after transfection, and the mRNA level of c-Jun was analyzed by qPCR. (**C**) CRL-2843 cells were transfected with *c-Jun* siRNA (60 nM), with NC as the control. The medium was changed at 12 h, followed by transfection with the nsp1 plasmid (1 μg). Cells were collected at 24 h, and the mRNA level of IL-1β was detected by qPCR. (**D**) Different doses of nsp1 plasmid (0.5, 0.75, and 1 μg/mL) were transfected into CRL-2843 cells, and the cells were collected at 24 h. The levels of p-c-Jun, c-Jun, Nsp1, and β-actin were analyzed by western blot. (**E and F**) CRL-2843 cells were transfected with *JNK* or *P38* siRNA (60 nM), with NC as the control. The medium was changed at 12 h, followed by transfection with the nsp1 plasmid (1 μg). The cells were harvested at 24 h. Western blotting was used to examine the levels of p-c-Jun, c-Jun, Nsp1, and β-actin. Differences were evaluated by Student's *t-*test. *, *P <* 0.05; **, *P <* 0.01.

To verify the role of AP-1 in nsp1-induced IL-1β production, we treated CRL-2843 cells with AP-1 inhibitor SR-11302, followed by transfection of nsp1. At 24 h post-transfection, we harvested the cells to analyze IL-1β mRNA level using qPCR. The results showed that the addition of AP-1 inhibitor significantly inhibited IL-1β expression induced by nsp1 in a dose-dependent manner (Fig. 4A). C-Jun is an important component of the transcription factor AP-1. We then designed and synthesized specific siRNA for *c-Jun* knockdown. The designed siRNA significantly knocked down c-Jun (Fig. 4B). Accordingly, AP-1 silencing led to a significant reduction in IL-1β expression induced by nsp1 (~65% decreases) (Fig. 4C). These findings indicate that AP-1 is the key transcription factor for nsp1 to induce IL-1β expression.

Next, we investigated whether c-Jun was activated by nsp1 in CRL-2843 cells. CRL-2843 cells were transfected with different amounts (0.5, 0.75, or 1 μg) of nsp1 for 24 h. Then, we examined the effect of nsp1 on c-Jun activation using a western blot. The results showed that the level of phosphorylated c-Jun was significantly increased in a dose-dependent manner, indicating that c-Jun is activated by nsp1 (Fig. 4D). Previous results have shown that AP-1 is a molecular protein downstream of the MAPK signaling pathway (16). To examine whether the JNK/p38 signaling pathway is essential for AP-1 phosphorylation induced by nsp1, we transfected CRL-2843 cells with different amounts (30 or 60 nM) of siRNAs for *JNK* or *p38* for 12 h, followed by transfection with the nsp1 expression plasmid. As shown in Fig. 4E and F, nsp1-induced phosphorylation of AP-1 was remarkably suppressed when *JNK* or *p38* was silenced. These data suggest that the nsp1-induced AP-1 activation is mediated by JNK and p38.

### PRRSV nsp1 activates JNK and P38 signaling pathways through TAK1 and TRAF6

**Fig 5:**
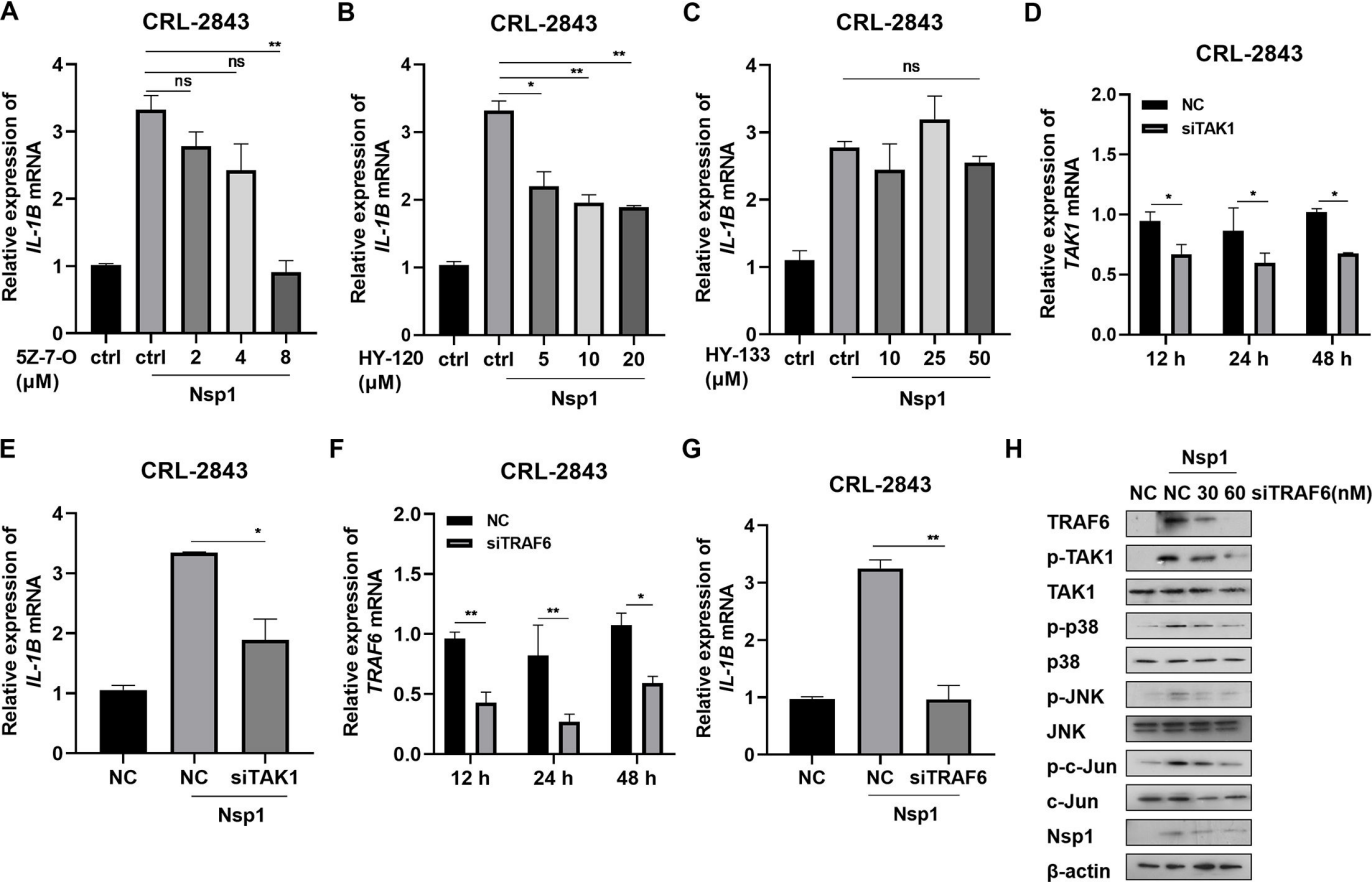
PRRSV nsp1 activates JNK and P38 signaling pathways through TAK1 and TRAF6. (**A–C**) CRL-2843 cells were pretreated with the TAK1 inhibitor (5Z-7-Oxozeaenol), TRAF6 inhibitor (HY-120934), or IRAK inhibitor (HY-13329) at different doses, and 1 h later, the cells were transfected with nsp1 plasmid (1 μg). The total RNAs were extracted at 24 h post-transfection for IL-1β mRNA analysis by qPCR. (**D and F**) CRL-2843 cells were transfected with *TAK1* or *TRAF6* siRNA (60 nM), with NC as the control. Cells were collected 12, 24, and 48 h after transfection, and the mRNA level of TAK1 or TRAF6 was analyzed by qPCR. (**E and G**) CRL-2843 cells were transfected with *TAK1* or *TRAF6* siRNA (60 nM), with NC as the control. The medium was changed at 12 h, followed by transfection with the nsp1 plasmid (1 μg). Cells were collected at 24 h, and the mRNA level of IL-1β was analyzed by qPCR. (**H**) CRL-2843 cells were transfected with *TRAF6* siRNA (30 or 60 nM), with NC as the control. The medium was changed at 12 h, followed by transfection with the nsp1 plasmid (1 μg). The cells were harvested at 24 h for western blot analysis. Differences were evaluated by Student's *t-*test. *, *P <* 0.05; **, *P <* 0.01; ns, not significant.

It is reported that TAK1, TRAF6, and IRAK are the important upstream mediators in JNK and p38 pathways (11). To further investigate the mechanism by which nsp1 activates the JNK/p38 pathway, we treated CRL-2843 cells with different doses of TAK1 (5Z-7-Oxozeaenol, 2, 4, and 8 μM), TRAF6 (HY-120934, 5, 10, and 20 μM), and IRAK (HY-13329, 10, 25, and 50 μM) inhibitors for 1 h, followed by transfection with the nsp1 expression plasmid. At 24 h post-transfection, the cells were harvested for qPCR analysis. The results showed that both the TAK1 inhibitor and the TRAF6 inhibitor significantly inhibited IL-1β expression induced by nsp1 in a dose-dependent manner (Fig. 5A and B). However, the IRAK inhibitor (HY-13329) had no significant effect on IL-1β production induced by nsp1 (Fig. 5C). These data suggest that nsp1-induced-IL-1β expression is dependent on TRAF6 and TAK1 signaling.

To further determine the role of TAK1 and TRAF6 in the production of IL-1β induced by nsp1, we designed and synthesized the siRNA for *TAK1* or *TRAF6*. The results showed that the designed siRNAs efficiently knocked down *TAK1* (Fig. 5D) and *TRAF6* (Fig. 5F), respectively. Then, we transfected CRL-2843 cells with the siRNA for *TAK1* or *TRAF6* for 12 h, followed by transfection with the nsp1 expression plasmid for 24 h. The qPCR analysis showed that *TAK1* or *TRAF6* silencing by siRNA led to a significant reduction of IL-1β induced by nsp1 (~43.6 and 70.3% decreases, respectively) (Fig. 5E and G). Next, to examine whether TRAF6 is essential for TAK1, JNK, p38, and AP-1 phosphorylation induced by nsp1, we transfected CRL-2843 cells with different amounts (30 or 60 nM) of siRNA for *TRAF6* for 12 h, followed by transfection with the nsp1 expression plasmid for another 24 h. As shown in Fig. 5H, nsp1-induced phosphorylation of p38, JNK, and AP-1 was remarkably repressed by silencing *TRAF6*. These data indicate that TAK1 and TRAF6 are required for nsp1 to activate the JNK/p38/AP-1 pathway.

### PRRSV nsp1 interacts with TRAF6

**Fig 6:**
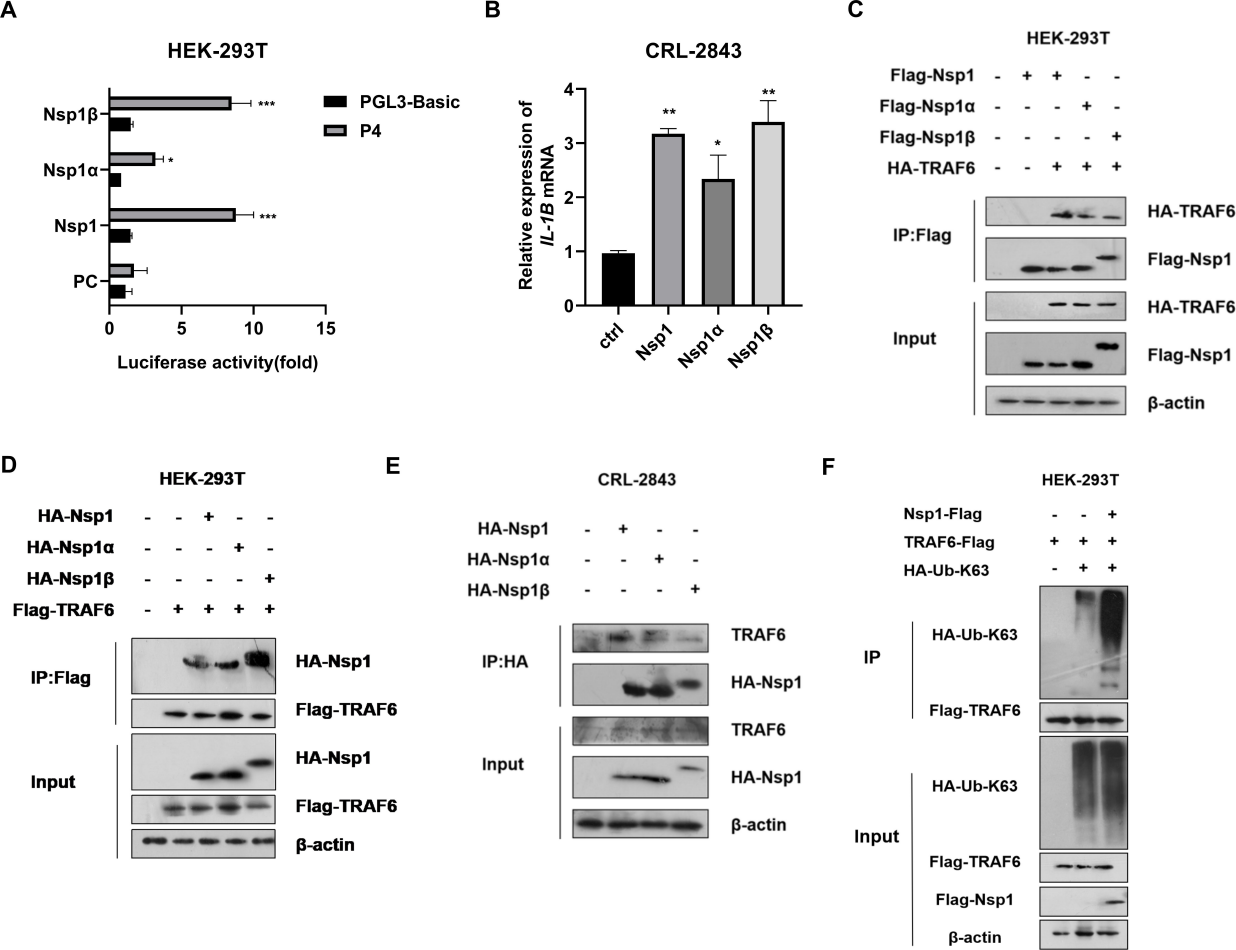
PRRSV nsp1 interacts with TRAF6. (**A**) HEK-293T cells were cotransfected with P4-luc promoter (0.5 μg) and Nsp1, Nsp1α, or Nsp1β plasmid (0.5 μg). The cells were harvested at 24 h post-transfection, and the luciferase activity was analyzed. (**B**) Nsp1, Nsp1α, or Nsp1β plasmid (1 μg) was transfected into CRL-2843 cells, and the IL-1β mRNA was analyzed by qPCR. (**C**) HEK-293T cells were cotransfected with Flag-Nsp1, Nsp1α, or Nsp1β (1 μg), and HA-TRAF6 plasmids (1 μg). The cells were harvested at 24 h later for Co-IP analysis. (**D**) HEK-293T cells were cotransfected with HA-Nsp1, Nsp1α, or Nsp1β (1 μg) and Flag-TRAF6 plasmids (1 μg). The cells were harvested at 24 h later for Co-IP analysis. (**E**) CRL-2843 cells were cotransfected with HA-Nsp1, Nsp1α, or Nsp1β plasmid (2 μg). The cells were harvested 24 h later for Co-IP analysis. (**F**) HEK-293T cells were cotransfected with Flag-Nsp1 (0.5 μg), Flag-TRAF6 (0.5 μg), and HA-Ub-k63 (1 μg). After 24 h, cells were treated with MG132 (20 μM) for 4 h, and then, the cells were harvested at 24 h later for Co-IP analysis. The ubiquitination levels of TRAF6 protein in IP and input were detected by western blot. Differences were evaluated by Student's *t*-test. *, *P <* 0.05; **, *P <* 0.01; ***, *P <* 0.001.

PRRSV nsp1 consists of 383 amino acids and has papain-like cysteine protease (PLP) activity. Nsp1 is usually processed into two multifunctional subunits: nsp1α (1–180 aa) and nsp1β (180–383 aa) (17). To investigate whether the two subunits of nsp1 are both involved in nsp1-induced IL-1β production, we transfected the IL-1β P4-luc promoter plasmid into HEK-293T cells with nsp1, nsp1α, or nsp1β plasmid to analyze the luciferase activity. As shown in Fig. 6A, either nsp1, nsp1α, or nsp1β significantly enhanced IL-1β promoter activity. To validate this result, we transfected CRL-2843 cells with nsp1, nsp1α, or nsp1β plasmid for 24 h. The qPCR analysis showed that nsp1, nsp1α, and nsp1β significantly induced IL-1β production, respectively (Fig. 6B).

Our results suggest that the target site for nsp1 to regulate the TRAF6/TAK1/JNK/p38/AP-1 signaling pathway is TRAF6. Thus, we investigated whether TRAF6 directly interacted with nsp1 and its subunits. We transfected Flag-Nsp1, Nsp1α or Nsp1β, and HA-TRAF6 plasmids into HEK-293T cells for 24 h before coimmunoprecipitation. As shown in Fig. 6C, nsp1, nsp1α, and nsp1β all interacted with TRAF6. We also performed a reverse co-IP experiment to confirm their interactions. HA-Nsp1, Nsp1α or Nsp1β, and Flag-TRAF6 plasmids were cotransfected into HEK-293T cells, and the results indicated that nsp1, nsp1α, and nsp1β interacted with TRAF6, respectively (Fig. 6D). To investigate whether nsp1 interacts with endogenous TRAF6, we transfected HA-Nsp1, Nsp1α, or Nsp1β expression plasmids into CRL-2843 cells and then harvested the cells for co-IP with anti-HA beads 24 h later. The results showed that nsp1 and its subunits all interacted with intracellular TRAF6 (Fig. 6E). Collectively, our data indicate that PRRSV nsp1, nsp1α, and nsp1β all interact with TRAF6.

Since the K63 ubiquitination of TRAF6 is important for activating downstream TAK1/JNK/p38, we subsequently investigated the effect of nsp1 on the ubiquitination of TRAF6. We co-transfected Flag-nsp1, Flag-TRAF6, and ubiquitin expression plasmids labeled with HA in HEK-293T cells. After 24 h, cells were treated with the proteasome inhibitor MG132 for 4 h. The cells were collected, and the effect of nsp1 on TRAF6 ubiquitination was detected. The results showed that nsp1 promoted the K63 ubiquitination of TRAF6 (Fig. 6F).

### Nsp1 R308 and R375 are the key amino acids for nsp1 to interact with TRAF6 and induce IL-1β production

**Fig 7:**
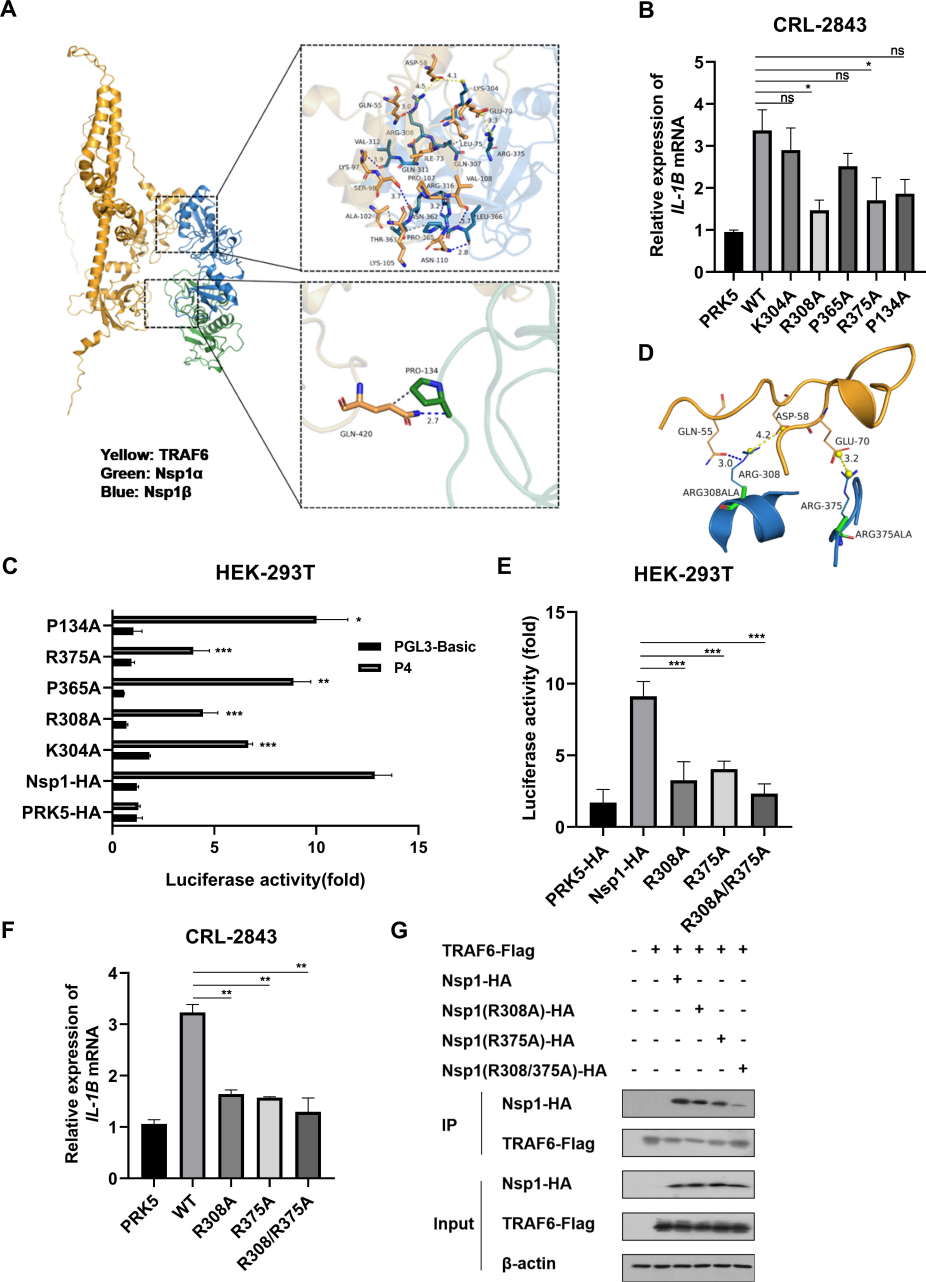
Nsp1 R308 and R375 are the key amino acids for nsp1 to interact with TRAF6 and induce IL-1β production. (**A**) Model for the predicted target sites on nsp1 that might interact with TRAF6, including P134, K304, R308, P365, and R375. (**B**) CRL-2843 cells were transfected with nsp1 or each of the five mutants with the mutated amino acid at P134, K304, R308, P365, or R375 (1 μg). The IL-1β mRNA was quantified by qPCR. (**C**) HEK-293T cells were cotransfected with nsp1 or its five mutants and the P4 promoter. The cells were harvested at 24 h post-transfection, and the luciferase activity was analyzed. (**D**) Model for the mutant with mutations at both R308 and R375. (**E**) HEK-293T cells were cotransfected with wild-type nsp1, R308, R375, or R308/375 and the P4 promoter. The cells were harvested at 24 h post-transfection, and the luciferase activity was analyzed. (**F**) CRL-2843 cells were transfected with nsp1, R308, R375, or R308/375 (1 μg), and the IL-1β mRNA was quantified by qPCR. (**G**) HEK-293T cells were cotransfected with Flag-TRAF6 vector and nsp1 (R308A)-HA, nsp1 (R375A)-HA, nsp1 (R308/375A)-HA mutants, or nsp1-HA. The cells were harvested at 24 h post-transfection for Co-IP analysis. Differences were evaluated by Student's *t*-test. *, *P <* 0.05; **, *P <* 0.01; ***, *P <* 0.001; ns, not significant.

To determine the important domain for nsp1 to induce IL-1β production, we constructed a series of truncated nsp1α and nsp1β using the luciferase reporter plasmid pGL3. Next, we co-transfected HEK-293T cells with IL-1β promoter and truncated nsp1α/β to analyze the luciferase activity. We found that the 67–180 aa of nsp1α and 81–203 aa of nsp1β contained essential amino acids for nsp1 to activate IL-1β promoter (data not shown). Next, to determine which amino acid(s) in nsp1 are important for nsp1 to interact with TRAF6 and IL-1β production, we used Alphafold2 software to analyze the interaction between nsp1 and TRAF6 in 3D structures. The results showed that five amino acids in nsp1 could be essential for the interaction of nsp1 and TRAF6: P134 (134 aa on nsp1α), K304 (124 aa on nsp1β), R308 (128 aa on nsp1β), P365 (185 aa on nsp1β), and R375 (195 aa on nsp1β), which may form salt bridges and hydrogen bonds with amino acids in TRAF6. These amino acids are in 67–180 aa on nsp1α and 81–203 aa on nsp1β. Then, we constructed five mutants, in which P134, K304, R308, P365, and R375 were replaced by alanine, respectively (Fig. 7A). Each of the mutant vectors was transfected into CRL-2843 cells for 24 h, and then, the mRNA level of IL-1β was analyzed by qPCR. Our results showed that nsp1 mutated at R308, R375, or P134 failed to induce IL-1β production, but the mutant at K304 or P365 did not (Fig. 7B), implying that R308, R375, and P134 aa are important for nsp1 to induce IL-1β production. To further elucidate the role of these amino acids (P134, K304, R308, P365, and R375) in activating the IL-1β promoter, we transfected HEK293T cells with the IL-1β P4 promoter plasmid and each of the nsp1 mutant vectors for 24 h. Luciferase assay showed that nsp1 mutated at K304, R308, or R375 failed to activate IL-1β promoter (Fig. 7C). These results indicate that R308 and R375 aa, but not P134 aa, are more important for nsp1 to induce IL-1β production and activate IL-1β promoter. Afterward, we constructed another nsp1 mutant (R308/375A), in which both R308 and R375 were replaced by alanine (Fig. 7D). Compared with the mutants of R308A or R375A, the mutant of R308/375A had less ability to activate the IL-1β promoter and IL-1β production (Fig. 7E and F). In addition, nsp1 mutants of R308A and R375A had impaired ability to interact with TRAF6 (Fig. 7G). Taken together, these data suggest that nsp1 R308 and R375 are the key amino acids for nsp1 to interact with TRAF6 and induce IL-1β production.

### Nsp1 R308 and R375 are essential for HP-PRRSV replication and inflammation

**Fig 8:**
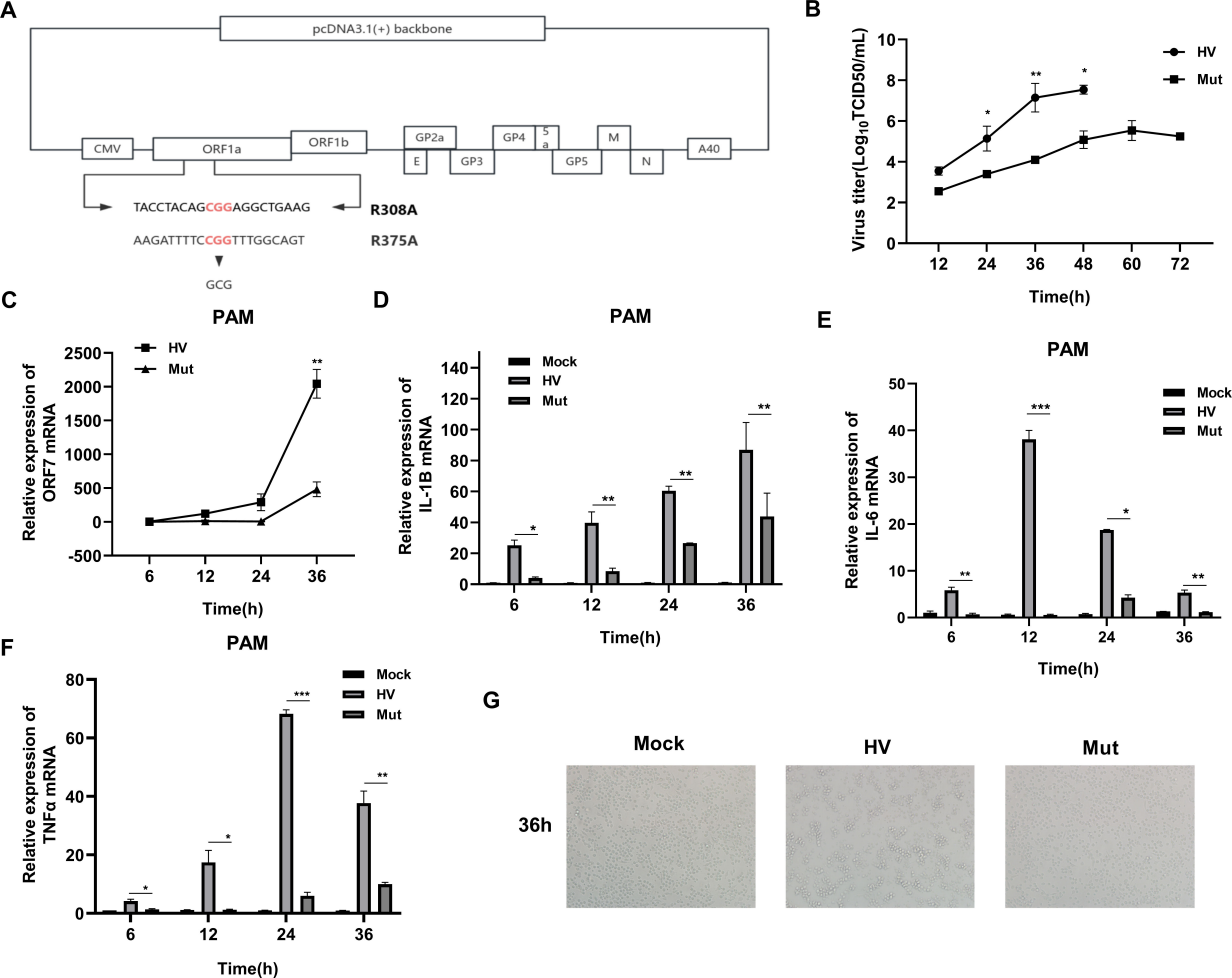
Nsp1 R308 and R375 are essential for HP-PRRSV replication and inflammation. (**A**) A mutant HP-PRRSV containing R308A and R375A mutations was constructed and named Mut. (**B**) PAMs were inoculated with the wild-type HP-PRRSV strain HV and the mutant virus PRRSV-R308/375A (Mut) at an MOI of 0.01, respectively. The cell supernatants were collected at 12 h, 24 h, 36 h, 48 h, 60 h, and 72 h post-inoculation, and the virus titer was detected. (**C–F**) Cells were harvested at the corresponding time points, and the mRNA levels of ORF7, IL-1β, IL-6, and TNFα were detected by qPCR. (**G**) PAMs were inoculated with HP-PRRSV strain HV and the mutant virus Mut (PRRSV-R308/375A) at an MOI of 0.01, respectively, and cell cytopathic effects were observed for 36 h post-infection. Differences were evaluated by Student's *t*-test. *, *P <* 0.05; **, *P <* 0.01; ***, *P <* 0.001.

Given the important roles of R308 and R375 in the nsp1-induced IL-1β production, we next tried to investigate their roles in HP-PRRSV-induced IL-1β production. We constructed the mutant virus PRRSV-R308/375A with mutations at R308 and R375 (Fig. 8A). To characterize the mutant virus, we inoculated PAMs with wild-type virus HP-PRRSV and the mutant virus PRRSV-R308/375A, respectively. Then, we collected the cell supernatants at different time points post-infection to analyze the virus titers using the TCID50 assay. The results showed that the replication rate of the mutant virus PRRSV-R308/375A was significantly lower than the wild-type HP-PRRSV (~10^2^ TCID50/mL decrease) (Fig. 8B), and qPCR analysis showed that the mRNA level of ORF7 was decreased about 76.4% (Fig. 8C). At the same time, the cells were collected at the corresponding time points to analyze IL-1β, IL-6, and TNFα expressions by qPCR. Compared with wild-type HP-PRRSV infection, the mRNA levels of IL-1β, IL-6, and TNFα were significantly decreased in PAMs infected with the mutant viruses PRRSV-R308/375A (~57.4%, 77.3%, and 91.2% decreases at 24 h, respectively) (Fig. 8D through F). Interestingly, IFNβ was significantly induced by the mutant virus PRRSV-R308/375A compared to the wild-type HP-PRRSV (data not shown). These data suggest that compared with the wild-type HP-PRRSV, the mutant virus PRRSV-R308/375A not only has a significantly lower replication rate *in vitro* but also has an impaired ability to induce excessive inflammation. To compare the cytopathogenic effects (CPE) caused by viruses, we inoculated PAMs with HP-PRRSV and the mutant virus PRRSV-R308/375A at an MOI of 0.01 for 36 h, respectively. Our results showed that PAMs infected with HP-PRRSV exhibited severe cell lysis, while the cells inoculated with the mutant virus PRRSV-R308/375A grew well with fewer lysed cells (Fig. 8G). Taken together, these data imply that R308 and R375 in nsp1 might be essential for HP-PRRSV replication and its ability to induce inflammation.

## DISCUSSION

Interstitial pneumonia is a common clinical feature of PRRSV infection in pigs (18, 19). Previous studies have shown that HP-PRRSV occurring in China and Southeast Asia has more severe interstitial pneumonia than classic North American PRRSV, which might be due to enhanced inflammatory immune response rather than higher levels of viral replication (20, 21). These observations highlight the important role of excessive inflammatory responses in HP-PRRSV infection and pathogenesis. Therefore, elucidating the underlying mechanisms of the inflammatory response will contribute to a deeper understanding of the infection and pathogenesis of PRRSV.

IL-1β plays a key role in immune responses, especially in initiating and regulating inflammatory responses. It has been reported that many viruses, such as Infectious Bursal Disease (IBD), Seneca Valley virus (SVV), and SARS-CoV-2, can upregulate IL-1β production (22–24). In the previous reports, PRRSV has been shown to upregulate IL-1β expression in PAMs and microglia cells (12, 25). However, the mechanism of IL-1β induced by PRRSV has not been clearly elucidated. Here, we confirmed that HP-PRRSV infection upregulated IL-1β production *in vitro* and then investigated the ability of HP-PRRSV proteins to induce IL-1β production. We found that nsp1 induced IL-1β production. The non-structural protein nsp1 is the first viral protein synthesized during PRRSV infection. Nsp1 has papain-like cysteine protease (PLP) activity and can auto-cleave itself to two subunits, nsp1α and nsp1β (17, 26). Among the non-structural proteins of PRRSV, nsp1 is considered to be the most effective protein in regulating the immune response of host cells (6). For example, nsp1α can inhibit IFNβ expression by degrading CREB-binding protein (CBP) and inhibiting IFN enhancer formation (27). Nsp1β degrades karyopherin-1 (KPNA1), which is known to mediate the nuclear import of ISGF3 (interferon-stimulated gene factor 3) (28). Nsp1α also reduces NF-κB activation (29). Nsp1β interrupts the phosphorylation of STAT1 and the nuclear translocation of ISGF3 of the JAK (Janus kinase)-STAT (signal transducer and activator of transcription) pathway (30). There is also a report showing that nsp1α and nsp1β are involved in the suppression of TNFα promoter activity through inhibiting the NF-κB activation and Sp1 transactivation (31). In consistent with the previous reports, we showed that NF-κB inhibitor (BAY11-7082) did not inhibit IL-1β expression induced by nsp1 in Fig. 2A. Our data also indicated that NF-κB was not the key regulatory element for nsp1 to induce IL-1β in Fig. 3B. However, we found that nsp1 and its two subunits nsp1α and nsp1β all induced IL-1β production through AP-1, emphasizing the important role of nsp1 played in modulating host innate immune response.

Mitogen-activated protein kinase (MAPK) is closely related to the occurrence of inflammation, tumors, and other diseases. The MAPK signaling mainly includes four pathways: ERK, JNK/SAPK, p38 MAPK, and ERK5/BMK1 (32–34). It has been reported that MAPK has been shown to be involved in the production of IL-1β induced by PRRSV (12), and PRRSV infection can activate MAPK p38, ERK1/2, and JNK in PAMs and Marc-145 cells (34–36). In the present study, we identified that nsp1 could activate MAPK pathways. By using different inhibitors of MAPK protein, we found that IL-1β expression induced by nsp1 was significantly decreased when JNK or p38 protein was inhibited. The same results were found when siRNA was used to knock down *JNK* or *p38*. In addition, western blot analysis showed that PRRSV nsp1 could activate the phosphorylation of JNK and p38. Thus, JNK and p38 are associated with PRRSV nsp1 induced IL-1β expression.

IL-1β expression is regulated by multiple factors, including NF-κB and AP-1 (12). To further investigate the transcriptional regulation mechanism of IL-1β induced by nsp1, we cloned the promoter of porcine IL-1β. We showed that using the inhibitor of AP-1 or silencing *AP-1* by siRNA downregulated the production of IL-1β induced by nsp1. Our western blot analysis also showed that nsp1 activated the phosphorylation of AP-1, and nsp1-induced phosphorylation of AP-1 was remarkably suppressed by silencing *JNK* or *p38*, implying that AP-1 is a key transcription factor that regulates IL-1β expression. Our data are consistent with the previous reports that IL-1β is directly regulated by the JNK/p38 MAPK pathway (37, 38). Extensive studies have revealed that TRAF6 and TAK1 are upstream of the JNK/p38 signaling pathways (39). TRAF6 is an E3 ubiquitin ligase that catalyzes the synthesis of polyubiquitin chains (PolyUb), and activates the mitogen-activated protein kinase (MAPK) signaling pathway to trigger proinflammatory transcription factor NF-κB and AP-1 activation (40). We found that the production of IL-1β induced by nsp1 was significantly decreased when TRAF6 or TAK1 inhibitors were added or *TRAF6* and *TAK1* were knocked down by siRNA. Accordingly, silencing *TRAF6* repressed nsp1-induced phosphorylation of p38, JNK, and AP-1. However, IRAK1 inhibitor has no effect on IL-1β expression induced by nsp1. These results indicate that TRAF6 and TAK1 are the upstream factors required for nsp1 to activate the JNK/p38/AP-1 signaling pathway.

Many viral proteins interact with MAPK signaling molecules to regulate inflammation. For example, the F317L protein of ASFV interacts with IκB kinase β (IKKβ) to inhibit its phosphorylation, resulting in a decrease in the expression of proinflammatory cytokines. The Nsp6 and ORF7a proteins of SARS-CoV-2 activate NF-κB through ubiquitination modification, thereby promoting the production of inflammatory cytokines (41, 42). This led us to speculate that nsp1 induces IL-1β through its interaction with MAPK signaling molecules. In our study, several lines of evidence suggest that nsp1 targets the TRAF6 protein. We performed co-IP assays to identify whether TRAF6 interacts with nsp1. The results showed that TRAF6 and nsp1 interacted directly. We found that nsp1 could promote the K63 ubiquitination of TRAF6 and thereby activate the downstream signaling pathway. We then analyzed the interaction between nsp1 and TRAF6 in 3D structures using Alphafold2 software and found five important amino acid sites. Among them, mutations of R308A and R375A in nsp1 impaired the interaction between nsp1 and TRAF6 and its ability to induce IL-1β. These data imply that nsp1 regulates IL-1β production by interacting with TRAF6, in which R308 and R375 play critical roles.

HP-PRRSV can cause stronger inflammatory immune responses, thereby inducing more severe symptoms in pigs. Since the two amino acid sites R308 and R375 are critical for nsp1 to induce inflammation, we then investigated whether R308 and R375 mutations had negative effects on HP-PRRSV-induced IL-1β production. Indeed, the mutant virus PRRSV-R308/375A with mutations in nsp1 had an impaired ability to replicate and induce more excessive inflammation than the wild-type strain HP-PRRSV in PAMs. In a previous study, mutation of 126–135 aa on PRRSV nsp1β is shown to contribute to the lower replication rate *in vitro* (43). Interestingly, we here also demonstrate that mutation in R308 (128 aa on nsp1β) and R375 (195 aa on nsp1β) resulted in decreased replication rates. Moreover, PRRSV-R308/375A infection can induce more IFNβ (data not shown). Thus, we assume that nsp1 mutation affects its ability to influence IL-1β and IFN-1, resulting in the diminished viral virulence. Previous studies have shown that the R308 and R375 sites in nsp1 are highly conserved in PRRSV strains (17), suggesting that these two amino acids might play an important role in PRRSV pathogenicity.

**Fig 9:**
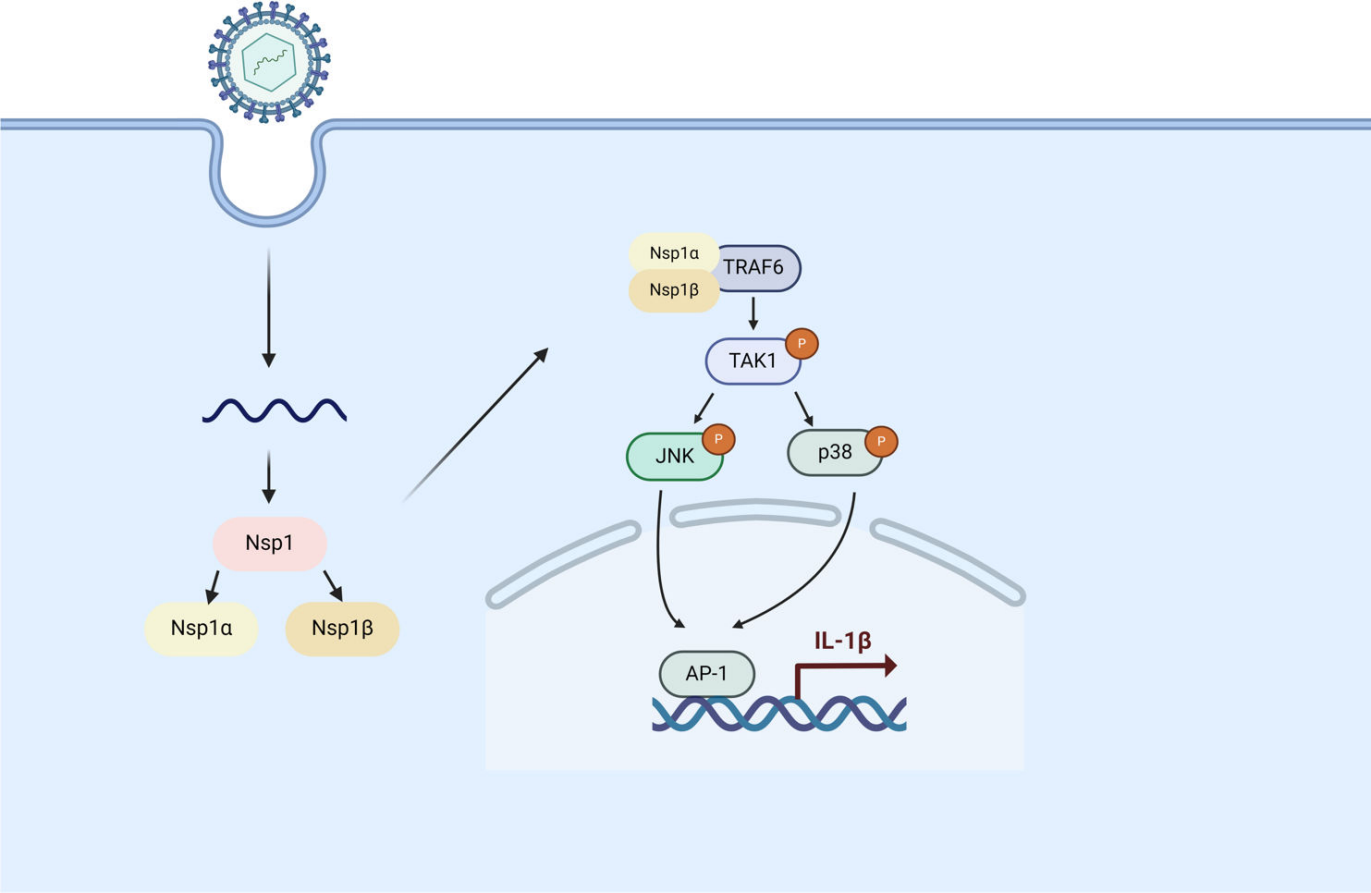
Model for HP-PRRSV nsp1 to induce IL-1β production. After HP-PRRSV infection, the viral protein nsp1 induces the expression of IL-1β via interacting with TRAF6 to activate the TAK1/p38/JNK/AP-1 signaling pathway, in which nsp1 R308 and R375 are the key amino acids for nsp1 to induce IL-1β production.

In this study, we investigated the underlying mechanisms of HP-PRRSV to induce IL-1β expression (Fig. 9). We confirmed that HP-PRRSV nsp1 is able to upregulate IL-1β production via the activation of TRAF6/TAK1/JNK/p38/AP-1 pathways, in which nsp1 R308 and R375 are the key amino acids for nsp1 to induce IL-1β production. Importantly, we successfully constructed the mutant virus PRRSV-R308/375A, which has a lower replication rate and impaired ability to induce excessive proinflammatory cytokines in PAMs. These findings will expand our knowledge on the understanding of HP-PRRSV pathogenicity and also give us some insights into the development of PRRSV vaccines.

## Data Availability

The data sets generated for this study are available on request to the corresponding author.
